# A Comprehensive Geriatric Workup and Frailty Assessment in Older Patients with Severe Aortic Stenosis

**DOI:** 10.3390/jcm13144169

**Published:** 2024-07-16

**Authors:** Enrico Brunetti, Fabiana Lucà, Roberto Presta, Niccolò Marchionni, Alessandro Boccanelli, Andrea Ungar, Carmelo Massimiliano Rao, Nadia Ingianni, Maddalena Lettino, Donatella Del Sindaco, Adriano Murrone, Carmine Riccio, Furio Colivicchi, Massimo Grimaldi, Michele Massimo Gulizia, Fabrizio Oliva, Mario Bo, Iris Parrini

**Affiliations:** 1 Geriatric Unit, Department of Medical Sciences, University of Turin, Hospital Città della Salute e della Scienza di Torino, 10126 Turin, Italyroberto.presta@unito.it (R.P.); mario.bo@unito.it (M.B.); 2 Department of Experimental and Clinical Medicine, University of Florence, Largo G. Brambilla 3, 50134 Florence, Italy; 3 Cardiology Department, Grande Ospedale Metropolitano di Reggio, 89124 Reggio Calabria, Italy; 4 UniCamillus University, 00131 Rome, Italy; 5 Cardiology ASP, 91100 Trapani, Italy; 6 Department for Cardiac, Thoracic and Vascular Diseases, Fondazione IRCCS San Gerardo dei Tintori, 20900 Monza, Italy; 7 UOC Santo Spirito-Nuova Regina Margherita-ASL Rome 1, 00153 Rome, Italy; 8 S.C. Cardiologia-UTIC, Ospedali di Città di Castello e di Gubbio-Gualdo Tadino, AUSL Umbria 1, 06127 Perugia, Italy; 9 Division of Clinical Cardiology, A.O.R.N. ‘Sant’Anna e San Sebastiano’, 81100 Caserta, Italy; 10 Clinical and Rehabilitation Cardiology Unit, San Filippo Neri Hospital, 00135 Rome, Italy; 11 Cardiology Department, Miulli Hospital, Acquaviva delle Fonti, 70021 Bari, Italy; 12 Cardiology Department, Garibaldi-Nesima Hospital, 95122 Catania, Italy; 13 Cardiovascular Department “A. De Gasperis”, ASST Niguarda Hospital, 20162 Milano, Italy; 14 Department of Cardiology, Mauriziano Hospital, 10128 Turin, Italy

**Keywords:** transcatheter aortic valve implantation, elderly, side adverse, frailty

## Abstract

Aortic stenosis (AS) represents a notable paradigm for cardiovascular (CV) and geriatric disorders owing to comorbidity. Transcatheter aortic valve replacement (TAVR) was initially considered a therapeutic strategy in elderly individuals deemed unsuitable for or at high risk of surgical valve replacement. The progressive improvement in TAVR technology has led to the need to refine older patients’ stratification, progressively incorporating the concept of frailty and other geriatric vulnerabilities. Recognizing the intricate nature of the aging process, reliance exclusively on chronological age for stratification resulted in an initial but inadequate tool to assess both CV and non-CV risks effectively. A comprehensive geriatric evaluation should be performed before TAVR procedures, taking into account both physical and cognitive capabilities and post-procedural outcomes through a multidisciplinary framework. This review adopts a multidisciplinary perspective to delve into the diagnosis and holistic management of AS in elderly populations in order to facilitate decision-making, thereby optimizing outcomes centered around patient well-being.

## 1. Introduction

Aortic stenosis (AS), including severe AS, is notably prevalent and increasingly incident in the older population [1,2]. While both surgical and transcatheter aortic valve replacement (SAVR/TAVR) are viable therapeutic options for patients with severe AS, TAVR has emerged as the primary choice for the vast majority of older patients with symptomatic severe AS, regardless of their surgical risk profile [3,4,5,6,7]. However, older patients with severe AS are very heterogeneous, including “fit and robust” subjects, as well as patients with different levels of cognitive and functional impairment, multimorbidity, polypharmacy, sarcopenia, and frailty [8]. It is well recognized that many elderly patients with poor health status and a high burden of physical and cognitive impairment may experience little or no benefit from TAVR [9,10]. Therefore, although TAVR is attractive due to its feasibility in most patients and its lesser morbidity, appropriate multidisciplinary decision-making, including a geriatric assessment, is crucial to estimate the expected net clinical benefit (survival with functional and symptomatic improvement and quality of life (QOL)), the risk of procedure-unrelated complications (e.g., delirium, prolonged hospital stay, functional decline, and institutionalization) as well as the best individual clinical pathway (e.g., nutritional support and physical pre-habilitation).

Compared to other research [11], this paper aims to delve deeper into TAVR indications in the elderly, featuring the most recent findings, cutting-edge research, and emerging trends in the field, including breakthroughs in tailored strategies based on a comprehensive, multi-integrated and multidisciplinary approach.

## 2. The Pre-Procedural Assessment of Older Patients

A careful multidisciplinary team (TMD) assessment of older patients with severe AS who are candidates for AVR is mandatory to evaluate the best individual procedural pathway to maximize benefits and reduce procedure-related complications. The STS score and EuroSCORE are frequently employed to anticipate patients’ outcomes for traditional cardiac surgical procedures. Nonetheless, surgical risk models have certain limitations and often inadequately predict clinical outcomes for TAVR [12,13]. While contemporary cardiac risk stratification often centers on predicting 30-day mortality and major adverse cardiac events as primary endpoints, it is crucial to acknowledge that for numerous older candidates undergoing TAVR, achieving functional independence and maintaining the ability to perform activities of daily living are their principal objectives [14]. Consequently, individual functional status may be particularly important for predicting outcomes in high-risk elderly candidates for SAVR/TAVR. These patients, particularly those aged over 80, comprise a diverse population: while some may be considered “fit and robust”, the majority contend with varying degrees of comorbidity burden, polypharmacy, functional and cognitive impairments, malnutrition, sarcopenia, and social isolation.

Consequently, it is not surprising that many of these TAVR patients, especially those aged over 80 years, face heightened risks of mortality, complications, and hospital readmissions [15,16]. Hence, there is a solid rationale for a geriatric assessment of elderly patients who are candidates for TAVR and geriatricians are involved in the MDTs to optimize these procedures [17,18]. This assessment should strive to distinguish patients for whom TAVR is likely to offer benefits from those for whom it may be futile or potentially detrimental. The latter group may experience prolonged hospital stays, lack of functional or QoL improvement, worsening cognition, and functional impairment due to hospital-associated complications such as bed rest, delirium, and sarcopenia. Additionally, it should help determine the optimal timing of the procedure and the most suitable individual clinical pathway for each patient.

The Comprehensive Geriatric Assessment (CGA) is essential for the holistic evaluation of elderly individuals from clinical, biological, and social standpoints, utilizing a combination of clinical scores, questionnaires, and tests to improve patient selection [8]. 

Clinical decisions should be tailored based on the patient’s risk category, expectations, and quality of life. Symptoms can be nonspecific or indicative of other underlying conditions. In cases where the severity of AS is uncertain, close monitoring is recommended, along with efforts to manage comorbidities and potentially refer the patient to rehabilitation to address frailty. If the risks outweigh the benefits, consideration of palliative care may be appropriate to improve the patient’s overall quality of life. In this context, an MDT, including geriatricians, must evaluate each patient for whom surgical intervention was deemed unsuitable to ascertain whether TAVR remains the optimal therapeutic strategy. This determination hinges not solely on chronological age but on a thorough and inclusive evaluation of the patient’s overall health status. The CGA also aids in preventing complications and enables prompt evaluation of periprocedural and postprocedural occurrences, improving patient selections. 

## 3. Imaging Evaluation

Echocardiography is the method of choice for assessing the severity of stenosis and can provide information on the morphology of the aortic valve (tricuspid or bicuspid). In the latter condition, the risk of implanting a pacemaker is greater. Additionally, echocardiography can evaluate the number of valve calcifications associated with a higher incidence of conduction disorders and perivalvular leaks. In elderly patients, there is a more frequent presence of more extensive atheroma that can lead to complications such as stroke, which is associated with greater calcification of the aortic valve.

Echocardiography can also provide helpful information about other heart valve diseases, left ventricular systolic and diastolic and right ventricular dysfunction. Severe mitral regurgitation could be the cause of subsequent episodes of heart failure (HF) [19,20,21]. In the pre-procedural multidisciplinary assessment phase, the balance between the geriatric evaluation and the echocardiographic examination contributes to delineating a clearer picture of the procedure’s future outcomes. 

Cardiac computed tomography (CCT) provides a comprehensive assessment of the aortic root, providing additional information on the feasibility of TAVR. In fact, a more accurate assessment of the dimensions of the aortic annulus and the distance between it and the coronary arteries helps reduce the risk of coronary artery obstruction [22]. Predominantly, women have a smaller aortic annulus, which may cause a high gradient. This mismatch can worsen hemodynamics and be associated with increased morbidity [23,24,25]. 

As age progresses, coronary artery disease (CAD) becomes increasingly prevalent, often leading patients to undergo conventional coronary angiography (CA). In cases where obstructive disease is identified, consideration should be given to percutaneous revascularization. The severity of CAD, particularly critical stenoses over 70% in the proximal coronary arteries, is associated with poorer survival rates following TAVR, indicating a higher risk profile for patients.

When assessing vascular access for TAVR procedures, femoral access is typically the primary choice, followed by subclavian (left or right transaxillary), common carotid, and brachiocephalic artery (i.e., retrograde access) if transfemoral access is not feasible. It is essential to note that both CT scans and coronary angiography require the administration of a contrast medium, which can cause acute kidney injury and contribute to short-term mortality in elderly patients.

## 4. Chronological and Biological Age

The threshold for entering old age is conventionally defined as 65 years [26]. Although this definition does not account for biological age, it is evident that factors such as comorbidity, frailty, cognitive deficits, social integration, and resource availability can significantly impact the evaluation beyond what demographic age alone might suggest [26]. Indeed, decline in physical and mental functions, reduced social interaction, and limited resources can affect mortality and QoL. The decline associated with biological aging is also influenced by genetic, biological, and environmental factors [11,27,28]. Therefore, differentiating biological aging from chronological aging plays a crucial role in patients’ selection and aids in more accurately stratifying patients who are candidates for TAVR.

Table 1 illustrates the principal studies that have included TAVR patients, categorized as elderly, with an average age between 73 and 83.

It is crucial to consider the fact that the initial randomized controlled trials (RCTs) comparing TAVR with SAVR in patients with severe AS at very high, high, and intermediate risk for early post-surgical mortality predominantly included octogenarians, while more recent RCTs conducted on low-risk patients have enrolled younger individuals with a mean age below 75 years [33]. This change in paradigm has led to prompt modifications to American [34] and European [35] guidelines, which now incorporate an age threshold to define suitable candidates for TAVI of >65 years in the United States and >75 years in Europe, irrespective of other pathological states, comorbidities, or frailty. Interestingly, no upper age limit has been suggested beyond which valve therapy should be withheld.

According to the registry’s data [36], advanced age is not a decisive factor in excluding patients from this therapeutic opportunity. Indeed, despite some evidence indicating higher in-hospital mortality among nonagenarian patients compared to younger ones [37,38], the observed-to-expected mortality ratio based on comorbidities and risk factors remained unchanged in the very elderly [39]. Therefore, these data suggest that meticulous patient selection remains necessary. 

Initially, TAVR procedures were performed with only a few types of devices. However, the increasing number of TAVR has driven significant advancements and diversification in device technology, leading to a broad range of options now available, such as smaller delivery sheaths and the ability to reposition or even fully recapture the device if it is not placed optimally. Moreover, the latest devices are smaller and aim to minimize residual aortic regurgitation’s incidence and severity. Currently, the choice of device is influenced by patient-specific anatomical factors (e.g., coronary height, peripheral vasculature dimensions), the operator’s preference, and the expertise of the medical center. The broader range of devices enhances the suitability of device selection for elderly individuals, including those with low body weight and small stature [40].

## 5. The Comprehensive Geriatric Assessment (CGA)

The CGA and its derived Multidimensional Prognostic Index (MPI) is the standard for multidimensional assessment to develop a coordinated plan to maximize overall health and to provide the best individual care in older persons [41,42]. This time-expensive assessment, requiring specific competence and skills, usually should be conducted by a geriatric team. Core components of the CGA (Table 2) include the assessment of comorbidities (Cumulative Illness Rating Scale, CIRS; Charlson Comorbidity Index, CCI) [43,44], polypharmacy, functional autonomy (Basic and Instrumental Activities of daily living, BADL and IADL, respectively) [45,46], cognition (Short Portable Mental Status Questionnaire (SPMSQ), Mini-Mental State Examination (MMSE) [47,48], mood (Geriatric Depression Scale, GDS), nutritional status (Mini Nutritional Assessment, MNA; Geriatric Nutrition Risk Index (GNRI) [49,50], sarcopenia-mobility (Short Physical Performance Battery, SPPB, including gait-speed and chair stand) [51,52], “frailty” and living situation and social support. There is robust evidence that such a granular approach to the older patient assessment may lead to improved outcomes including decreased hospitalization, nursing home admission, and mortality in several clinical settings [42,53,54,55,56].

A recent consensus statement has generated a document systematically analyzing the current evidence regarding the predictive efficacy of various tools, often grouped under the term “frailty”, on clinical outcomes associated with TAVR. These outcomes include mortality, neurological complications such as delirium leading to prolonged hospitalization, and quality of life factors such as discharge location and readmission rates [57]. After a careful review of the available evidence, the authors concluded that (i) the 5 m gait speed assessment is advised to predict perioperative and intermediate to long-term mortality, and the SPPB is advised to predict intermediate-term mortality; (ii) the GNRI is advised for the prediction of increased risk of prolonged hospitalization. While this approach is methodologically sound, it possesses a significant inherent limitation: focusing solely on individual aspects of the multidimensional assessment overlooks the comprehensive insight gained from evaluating multiple domains simultaneously [58]. Indeed, there is consistent evidence that the CGA may provide a more granular definition of the expected clinical benefit at an individual level [8,59,60,61,62,63,64,65], and the CGA-derived MPI has been shown to predict mortality and adverse clinical outcomes in elderly patients undergoing TAVR. [41,63,66]. Moreover, such a comprehensive assessment provides information about potential futility: of note, four CGA domains (advanced dementia, severe sarcopenia/cachexia, disability for all/most BADL, bed bound/poor mobility) are among the five risk factors for TAVR futility according to the 2019 Canadian Cardiovascular Society Position Statement TAVR [67]. Eventually, from a clinical point of view, this assessment may provide further information about the risk of common complications such as delirium, prolonged hospital stay/discharge in a long-term facility, functional decline, and/or no functional/quality of life benefit [8,65,68,69,70,71].

## 6. The Concept of Frailty and Frailty Tools 

**Figure 1:**
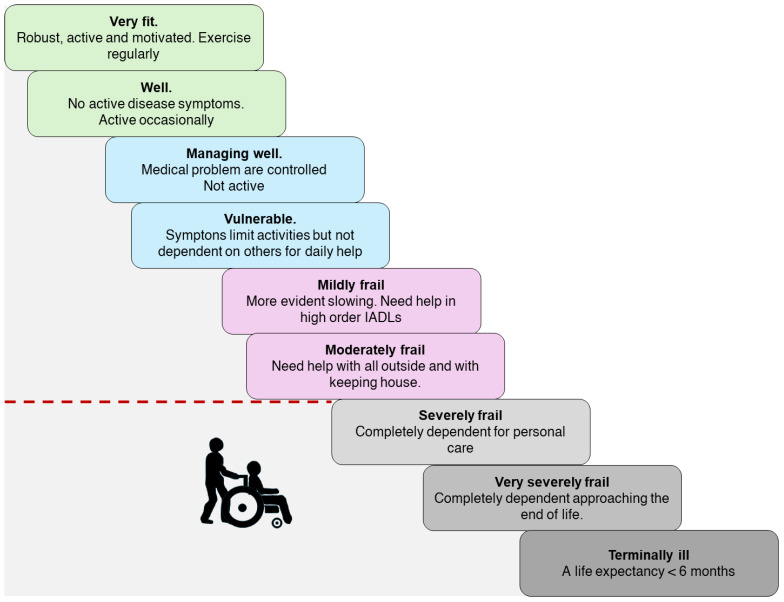
The Clinical Frailty Scale (CFS) is used to assess the degree of frailty in older adults. Frailty is a multidimensional syndrome characterized by a poor physiologic condition and increased vulnerability.

Regrettably, both the CGA and the MP, typically conducted by specialists in geriatrics, entail intricate and time-intensive procedures, thus constraining their application in routine clinical practice beyond geriatric contexts. Furthermore, implementing this multidimensional assessment may prove impractical in certain clinical settings lacking geriatric expertise. Pursuing a more manageable alternative to this intricate evaluation, “frailty” emerged as an appealing surrogate, garnering widespread endorsement among cardiologists and cardiac surgeons. Since frailty predisposes patients to higher mortality, morbidity, and impaired functional recovery, the frailty assessment has assumed a central role in the pre-procedural evaluation of older TAVR candidates. Although the definition of ‘frailty’ remains a controversial issue, it has been characterized as a clinical, multifactorial geriatric syndrome involving a multi-systemic impairment, resulting in decreased physiological reserves and increased susceptibility to stressors [72]. Since the inception of this foundational definition of frailty, the proliferation of frailty assessment tools has been steadily increasing. This proliferation, coupled with the absence of a unified consensus regarding frailty’s definition and measurement, has presented notable challenges and fostered considerable confusion, both within research endeavors and clinical environments [72,73]. The “ambiguity” surrounding the conceptualization of “frailty” was apparent in the international guidelines concerning [74]. Indeed, the 2017 ACC guidelines underscored the significance of assessing 5 min gait speed, disability in activities of daily living, cognitive impairment, depression, and malnutrition as key indicators of “frailty” that should be routinely evaluated before TAVR. Similarly, even broader recommendations were outlined in the European Guidelines [75]. Hence, in the age of precision medicine, providing a concise overview of frailty and the array of available assessment tools becomes imperative for a medical metric of such extensive application in clinical medicine and surgery. Despite the abundance of tools designed to gauge frailty, the majority stem from two primary conceptual frameworks. Initially, frailty was construed as a distinct physical dimension, typically antecedent to the onset of disability, albeit acknowledging the potential coexistence of these conditions [72]. In line with this perspective, Fried and collaborators delineated frailty as a syndrome characterized by a gradual age-related decline across multiple physiological systems, leading to heightened susceptibility to stressors and an elevated risk of unfavorable clinical outcomes. This syndrome is identified by at least three criteria, which may include slow gait speed, reduced levels of physical activity, unintentional weight loss, self-reported exhaustion, and muscle weakness [76]. This concept of the “frail phenotype” (FP) characterizes frailty as a singular domain within a broader multidimensional health evaluation. It does not inherently denote a high disease burden or the manifestation of functional dependence but is intricately linked with sarcopenia. The FP is correlated with deteriorating mobility and autonomy, increased hospitalizations, and elevated mortality rates over seven years among older individuals residing in the community [72,76]. Patients exhibiting FP may face heightened risks post cardiac surgery and cardiovascular interventions, as well as increased susceptibility to complications from medical treatments. This underscores the potential for a customized clinical approach to managing these patients. Nevertheless, diagnosing the FP during a busy clinical environment can prove challenging. Consequently, various alternative, more straightforward tools have been suggested for its identification, including the SPPB and the 5 m gait speed test [52,73]. Coinciding with this development, Rockwood and colleagues introduced an alternative perspective on frailty rooted in the multidimensional evaluation of the CGA. Their “deficit accumulation model” (DAM) posits that the greater the number of health deficits an individual exhibits—encompassing functional limitations, disabilities, cognitive and sensory impairments, psychosocial factors, and multiple diseases—the higher their risk for adverse outcomes [77]. The FI discerns frailty in older adults by assessing the proportion of deficits among 70 evaluated items (with several condensed versions available). Frailty severity is categorized as mild (FI-CGA 0–7), moderate (FI-CGA 7–13), and severe (FI-CGA ≥ 13). Elevated FI scores correlate strongly with heightened risks of short-term mortality and institutionalization, effectively identifying individuals with intricate health profiles and heightened vulnerability to short-term adverse *outcomes* [78]. Recognizing the time-intensive nature of the FI, Rockwood and colleagues validated the semi-quantitative 7-point scale known as the Clinical Frailty Scale (CFS). This scale employs a global “eyeball” vulnerability assessment, ideally derived from a CGA. Subsequently, the CFS was refined into the current inclusive 9-point scale, categorizing individuals as “very fit”, “fit”, “managing well”, “very mild frailty”, “mild frailty”, “moderate frailty”, “severe frailty”, “very severe frailty”, or “terminally ill”. Scores below 3 indicate non-frail patients, while scores ranging from 4 to 6 signify individuals with mild-to-moderate frailty. Scores exceeding 6 conventionally denote patients with severe frailty and poor life expectancy (see Figure 1) [79,80]. The CFS demonstrates a strong correlation with FI scores, with increasing CFS severity significantly linked to elevated risks of mortality and institutionalization [78,79]. Indeed, the CFS is widely used, but its reliance on subjective clinical judgment for scoring presents a challenge regarding inter-rater reproducibility. This limitation may impede its broader adoption, especially among less experienced raters [81].The existence of divergent conceptualizations of “frailty” can understandably lead to confusion among the public. While the FP isolates physical frailty as a single domain within a multidimensional assessment, both the FI and the CFS offer a broader evaluation of health status, encompassing factors such as disease burden, disability, cognitive and sensory impairments, and psychosocial variables (in the case of the FI) or relying on expert geriatric evaluation (in the case of the CFS). Additionally, while most FP models categorize individuals as frail or non-frail (and sometimes pre-frail), the FI and the CFS offer prognostic insights across varying levels of frailty severity [80]. Consequently, the clinical implications derived from these tools more closely resemble those provided by the Multidimensional Prognostic Index (MPI) than those associated with the FP.

Unsurprisingly, patients identified as “frail” using these different tools exhibit divergent prognoses. Indeed, the 2-year all-cause mortality rates among older patients classified as severely frail according to the FI/CFS (40–50%) markedly exceed those reported for subjects identified with the FP (around 10%) [73]. Indeed, an expanding array of “frailty” tools utilizing hospital or administrative electronic databases have been increasingly employed across various clinical settings. These tools often leverage electronic health records from community or hospital settings, integrating ICD-10 codes, resource utilization data, and a wide spectrum of acute and chronic health conditions (primarily cardiovascular), abnormal laboratory findings, indicators of healthcare service utilization, medical equipment usage, or a combination of signs and symptoms. However, it is notable that certain frailty risk scores derived from these tools demonstrate poor concordance with well-established assessment instruments, leading to varied prevalence figures for frailty reported in studies. Consequently, whether these diverse tools identify the same patient phenotype and, more critically, whether they can effectively assess biological age and predict residual life expectancy remains unresolved [73].

## 7. Frailty and Outcome

Indeed, numerous studies have consistently demonstrated that frailty is associated with heightened mortality rates and an increased likelihood of adverse outcomes. However, many frailty scores exhibit significant variability and yield divergent prevalence estimates depending on the specific frailty assessment tool utilized [82]. Nonetheless, consistent evidence indicates that validated frailty models hold strong predictive value in older patients considered for aortic valve replacement (AVR) or TAVR. Two distinct frailty indices, based on the definitions proposed by Fried and Rockwood (FP and FI, respectively), identified individuals at heightened risk of mortality and functional impairment among 1442 patients aged 65 years or older participating in the US CoreValve High Risk (HiR) or Surgical or Transcatheter Aortic-Valve Replacement in Intermediate Risk Patients (SURTAVI) trial [83]. The FP was associated with increasing mortality among very old patients with severe AS, irrespective of AS treatment [84], and with increased discharge to rehabilitation facilities among older patients who underwent TAVR [85]. In the OCEAN multicenter registry, which comprised patients undergoing TAVR, with approximately 50% aged 85 and older and 14% exhibiting moderate-to-severe CFS scores, the severity of frailty emerged as an independent predictor of heightened late cumulative mortality risk [86]. In both the OCEAN-TAVR and FRAILTY-AVR studies, increasing CFS scores, with a notable cutoff of around 6 points, exhibited a strong association with escalating mortality rates among older patients who had undergone aortic valve replacement (AVR) [87]. In a nationwide cohort study encompassing 28,531 patients evaluated using the Hospital Frailty Risk Score and undergoing TAVR, 1-year mortality rates were 7.6%, 17.6%, and 30.1% among those categorized with low-, medium-, and high-risk scores, respectively [31]. In a 4-year study involving patients undergoing TAVR, the Johns Hopkins Claims-based Frailty Indicator, designed to identify patients meeting the Frail Phenotype (FP) with a cutoff score greater than 0.11, was shown to predict adverse health outcomes. These outcomes included falls, deteriorating mobility, hospitalization, and mortality [31]. Increasing severity of frailty detected through the FI has been demonstrated to be associated with an increased risk of delirium among older hospitalized patients [88], and increasing severity of the pre-procedural FI score was associated with a lower probability of functional improvement and a greater probability of functional decline after SAVR/TAVR in older patients [9]. In a study comparing the predictive capabilities of the Frailty Index (FI) and the Frail Phenotype (FP) among older patients undergoing SAVR or TAVR, the deficit accumulation FI demonstrated superior prognostic accuracy in predicting death or poor recovery compared to the FP [89]. Furthermore, research has shown that the presence and severity of frailty in patients undergoing TAVR independently correlate with a substantial rise in hospitalization costs [90]. Among the various frailty assessment tools available, specific instruments have proven clinically valuable in the context of AS. The Essential Frailty Toolset (EFT), comprising albumin levels, presence of anemia, ability to perform chair raises, and Mini-Mental State Examination (MMSE) scores, demonstrated superior performance compared to previously described frailty scores in predicting mortality and worsening disability. Another simplified tool, incorporating serum albumin levels, 5 min gait speed, and the presence of anemia, was also shown to be associated with adverse clinical outcomes [87,91]. 

The Erasmus Frailty Score (EFS), a multidimensional frailty scale derived from a CGA, has been shown to correlate with an elevated risk of delirium, 1-year mortality, and poor outcomes among older patients undergoing TAVR [68]. 

## 8. Frailty and Futility

**Figure 2:**
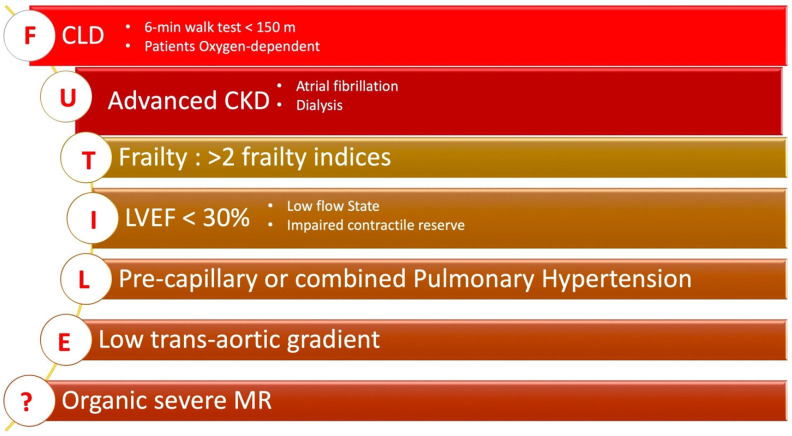
Factors of TAVR non-response. Abbreviation: CLD: chronic lung disease; CKD: chronic kidney disease; MR: mitral regurgitation; LVEF: left ventricle. Ejection fraction.

It is well recognized that a non-negligible proportion of patients do not fully benefit from TAVR despite a technically “perfect” procedure [92,93]. While the concept of futility in medicine lacks a uniform definition, within the context of TAVR, it typically encompasses a combination of non-cardiovascular-related mortality and/or lack of objective symptomatic improvement in the New York Heart Association (NYHA) functional class. Consequently, a comprehensive post-TAVR outcome measure has been suggested, incorporating both mortality and quality of life assessments within a unified composite endpoint [94]. Recently, subgroups of patients for whom TAVR might not have significant benefit have been identified [95] Figure 2.

### 8.1. Chronic Lung Disease (CLD)

Chronic lung disease (CLD), which occurs in 30% of TAVI candidates, has been demonstrated to be associated with worse clinical and functional post-TAVI outcomes. According to several data [96,97,98], CLD patients undergoing TAVI exhibit higher early mortality, with a 1-year mortality rate of 30% compared to a 1-year mortality rate of less than 20% in non-CLD patients. Therefore, assessing moderate-severe CLD as a marker of futility is crucial, based on a quantitative and functional evaluation of disease severity, such as an unsatisfactory six-minute walk test (6MWT). Approximately 75% of patients whose pre-TAVI 6MWT was <150 m died at follow-up compared to nearly 25% of patients whose baseline 6MWT was ≥150 m.

### 8.2. Chronic Kidney Disease (CKD) 

With advancing age, a progressive decline in renal function physiologically occurs [99]. Chronic kidney disease (CKD) has been reported in 30–50% of TAVR candidates [100]. The severity of CKD has been shown to be associated with both early and late mortality post TAVR [101], with a 1-year mortality rate exceeding 30% and a poorer prognosis in patients with severe CKD [102].

### 8.3. Pulmonary Hypertension (PH)

Pulmonary hypertension (PH) often occurs in patients with LV dysfunction [103]. A PH value ≥ 50 mmHg in patients undergoing TAVR has been proposed as an optimal cutoff to predict post-TAVI outcomes [103]. Higher 1-year post-TAVR mortality has been reported in patients with precapillary PH and combined precapillary and postcapillary PH [104]. The presence of combined PH has been shown to be a strong predictor of 1-year mortality. Additionally, RV function plays an important prognostic role in patients with PH [104].

### 8.4. Left Ventricular Dysfunction

A systolic left ventricular dysfunction often occurs in AS, with LVEF values ≤ 30% ≤ 50% of 6–11% and 27–46% of TAVI have been reported, respectively, in TAVI candidates [105]. According to some data, a baseline LVEF < 40% is associated with early mortality [106,107]. Conversely, a post hoc analysis of the PARTNER trial shows that a reduced LVEF does not influence the prognosis [108]. However, it should be considered that LVEF might not be an accurate marker of myocardial dysfunction in the presence of severe AS, while a low trans-aortic flow could be a more important prognostic factor [109].

### 8.5. Severe Mitral Regurgitation 

In TAVI candidates, the reported incidence of moderate-severe mitral regurgitation (MR) ranges from 2% to 33% [110], rising to 50% among TAVI patients with organic MR [111]. However, the influence of MR on TAVR outcomes remains debated. Some data suggest that moderate-severe MR adversely affects both short-term (30-day) and long-term survival; however, improvement in MR severity has been observed in 50% of patients with moderate-severe MR following TAVI [111]. A post hoc analysis of PARTNER data indicated that moderate-severe MR represented an unfavorable prognostic factor at 2 years after surgical valve replacement but not after TAVI [112]. In a meta-analysis involving over 8000 cases, moderate-severe MR was associated with increased mortality at 30 days and 1-year post-TAVR, despite improvement in 50% of patients. Organic MR is unlikely to decrease following TAVR [113].

### 8.6. Patients with a Life Expectancy of Less Than One Year

Patients with a “survival with benefit” probability of less than 25% at two years are defined as having an improvement of at least one New York Heart Association (NYHA) functional class and/or one Canadian Cardiovascular Society (CCS) class angina symptom and/or improvement in quality of life or life expectancy.

**Figure 3:**
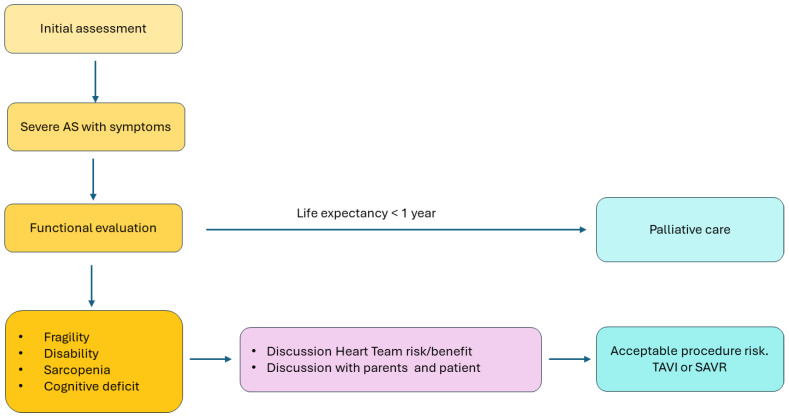
Clinical pathway of geriatric assessment in older patients with severe aortic stenosis.

However, measuring these endpoints can be challenging in older patients with multiple comorbidities and varying functional levels. A more comprehensive approach underscores the importance of individualized assessment to identify better patients for whom TAVR may be futile [114] (Figure 3).

The 2019 Canadian document on TAVR [67] recommended considering certain factors as indicators of potential futility. These include advanced dementia, severe sarcopenia/cachexia, disability in most or all basic activities of daily living (BADL), being bed-bound, or having poor mobility (beyond severe CKD). Similarly, Afilalo proposed “red flags” to alert cardiologists and cardiac surgeons to the potential high risk of procedure futility. These red flags include advanced dementia, advanced kidney or liver failure, inability to complete a short-distance gait speed test or chair-rise test, malnutrition, sarcopenia, or dependency on most basic activities of daily living [115]. While an “eyeball” evaluation may suffice for patients exhibiting specific clinical characteristics to warrant potentially denying the procedure, for most older patients with severe AS eligible for SAVR/TAVR, the use of validated assessment tools is recommended. In these cases, when feasible, a specialized CGA offers the most comprehensive approach to providing prognostic and treatment insights to the multidisciplinary heart team. In instances where this level of support is unavailable, physicians with some experience in the field should utilize validated frailty assessment tools.

## 9. The Cardiologist’s Role

TAVR has indications in elderly patients at high surgical risk with a life expectancy of more than one year because it further increases survival and improves quality of life. However, age alone is not a discriminating factor, while frailty and comorbidities are related to worse survival in patients with AS undergoing TAVR and more peri-procedural complications. 

Identifying cardiac and non-cardiac factors associated with negative outcomes following TAVR is imperative. CLD, CKD, and frailty have been suggested as predictors of the futility of TAVR due to poor post-procedural outcomes. However, it becomes complex for the clinician to deny a patient the opportunity for TAVR based on a single variable related to respiratory or renal function. Furthermore, the evaluation by a multidisciplinary team is essential for a comprehensive and holistic assessment of the patient, especially for more specific evaluations of frailty. 

Regarding cardiac conditions, available data suggest that reduced LVEF cannot be used as an isolated factor to determine post-TAVR futility. Instead, the presence of low flow, severe PH, especially pre-capillary or combined, and severe organic MR are CV factors that should be considered in the clinical decision-making process for potential TAVR candidates. Additionally, studies have demonstrated the negative impact of pre-existing or new-onset AF on post-TAVR morbidity and mortality. Indeed, AF itself is more likely a marker of underlying advanced heart disease such as HF, multivalvular disease, and more extensive vascular disease [21]. Furthermore, the management of TAVR in patients with AF is challenging due to the complexities associated with antithrombotic therapies, bleeding risks, and ischemic events. Therefore, it is unlikely that a single CV factor is sufficient to identify a group of patients for whom TAVR would be futile [116].

The heart valve clinic (HVC) approach has been recently proposed in clinical practice with the aim of managing people with moderate AS and asymptomatic severe AS in order to optimize the timing of TAVR [117,118], recognizing symptoms before the development of LV dysfunction [116].

Indeed, detecting changes in clinical status in the preoperative period is crucial for accurately evaluating operative risk [119,120].

HVC patients have been shown to undergo TAVR in an early stage, wasting less time from clinical evaluation to the procedure. This aspect assumes a pivotal role in managing these patients due to the fact that the risk of death accounts for surgery of about 15% per year [116,117].

In the natural history of AS, HF occurrence adversely impacts prognosis. Patients should be provided with the chance to optimize medical therapy. Therefore, HCV patients may benefit from comprehensive management, undergoing an accurate and timely evaluation.

Indeed, a lower risk of mortality, both CV and for all causes, has been reported in HCV patients [116,121,122].

Moreover, the evaluation of the HCV is strategic in excluding the group of patients for whom TAVR may not be advantageous or subject to the risk of the procedure’s futility [80]. 

The elderly patient approaches the risk of TAVR surgery with greater benefit when the surgery is preceded by a period of rehabilitation aimed at counteracting frailty.

A number of stratification models are available to assess cardiac surgical risk, including the EUROSCORE and Society of Thoracic Surgery (STS). However, these models underestimate risk because they do not take into account the frailty factor, while the SURTAVI takes frailty into account but still needs to be validated [74,123,124].

The European guidelines guide TAVR in elderly patients aged ≥ 75 years or below when there are comorbidities or they are judged inoperable. In comparison, the American guidelines set the limit at >65 years [24,125]. According to a study by Delijani et al. in 2022 based on the analysis of the United States National Readmission Database (NRD) on 84,017 patients undergoing TAVR from 2016 to 2018, patients > 80 years have a higher risk of readmission, complications, and mortality, while the 70–79 age group showed no significant differences compared to patients under 70 years old [15]. 

The Partern trial randomized 699 patients between surgical and transcatheter valve replacement and found that the two procedures had equivalent one-year survival rates despite an increased risk of major peri-procedural complications such as renal failure requiring dialysis and major vascular complications (perforation or dissection of the aorta) [7]. Consequently, the cardiologist must carefully assess the patient’s anatomy and the valve.

## 10. Post-Operative Phase

Complications related to vascular access, such as bleeding and pseudoaneurysms, are common in this frail and elderly population due to the use of large catheters. Additionally, persistent inter or intraventricular disturbances, which may necessitate permanent pacemaker implantation, can have significant implications in this demographic.

Periprosthetic complications, including the persistence of minor leaks around the valve, should also be considered. Furthermore, embolic events such as stroke, resulting from valve debris or vascular system origin, as well as the onset of atrial fibrillation (AF), can profoundly impact both quality of life and survival due to the associated risks of immobilization and cognitive impairment.

Balloon-expandable devices (e.g., Sapien 3 and Sapien 3 ultra-models) are used in dilated or angulated ascending aorta cases. These expand using their radial force by adapting to the oval annular morphology. Self-expanding valves (e.g., CoreValve, theirEvolut R, Portico, and Jena Valve models) expand directly into the aortic annulus [126].

Following TAVR, a seamless transition to post-procedural care is pivotal for patient recovery. This includes the crucial steps of evaluating regurgitation and monitoring for pericardial effusion via echocardiography. Concurrently, initiating antiplatelet therapy to prevent thrombosis is paramount. Given the lower bleeding risk, direct oral anticoagulants (DOACs) are preferred over vitamin K antagonists (VKAs) for patients with AF undergoing TAVR [127].

## 11. Post-Discharge Care

Early rehospitalization during the first year after discharge has been correlated with the procedure, whereas late rehospitalization is associated with comorbidities. Frailty after discharge is associated with increased mortality and morbidity. Therefore, frail patients are more likely to be transferred to a rehabilitation center after discharge [85,128]. 

Comprehensively instructing a caregiver at discharge can be beneficial because it allows the patient to be transferred home. Treatment of malnutrition and sarcopenia and careful adherence to medical therapy are desirable goals. The caregiver’s role is considered to be pivotal in postoperative care for managing the patient and therapy.

**Figure 4:**
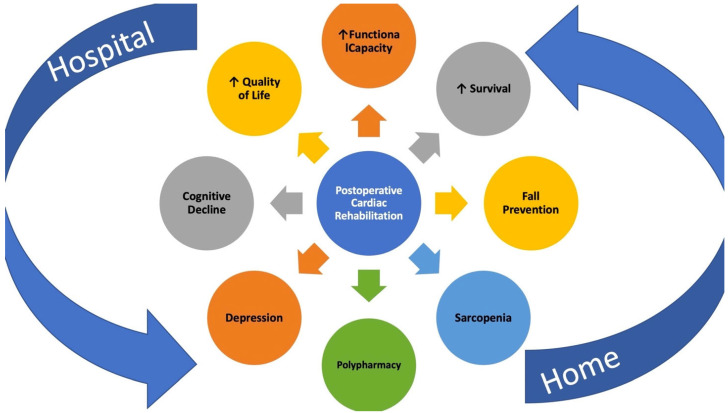
Cardiac rehabilitation (CR) after transcatheter aortic valve replacement (TAVR).

Furthermore, in elderly patients, postoperative rehabilitation (CR) is crucial to facilitating the transition from hospital to home or a care facility. CR after TAVR surgery not only enhances exercise capacity and survival but also addresses other geriatric-specific issues such as balance, fall prevention, sarcopenia, polypharmacy, depression, and cognitive decline [129]. The final objective of CR is to improve the quality of life (QoL) (Figure 4).

After TAVR surgery, CR aims to improve the patient’s physical and psychological status and overall well-being. This program often involves exercise training, education and counseling, monitoring and support, psychosocial support, and nutritional guidance. 

## 12. Conclusions

Current evidence suggests that older patients undergoing TAVR may benefit similarly to younger patients. However, the older population includes a marked heterogeneity in global health and functional status, implying that a non-negligible proportion of these patients might not derive survival, clinical, symptomatic, or functional benefit from a technically perfect procedure. An appropriate multidimensional assessment is mandatory to avoid futility and potential harm and to provide the best individual clinical decision-making. 

General surgical scores are not reliable in estimating long-term mortality and functional and symptomatic benefit in older TAVR patients, in whom the burden of comorbidities, the presence and severity of geriatric syndromes (cognitive impairment/dementia, basic and instrumental functional dependence, malnutrition, and sarcopenia, frailty and social isolation) are by far more reliable predictors of death and functional, symptomatic, and quality of life improvement after TAVR.

Appropriate patient selection and careful procedural planning, including the choice of the appropriate access route, technique, and type and size of the TAVR valve, and adequate postoperative care, including appropriate rehabilitation measures and drug therapy [130], all contribute to reducing the mortality risk of TAVR. In experienced hands, the CGA and some validated frailty tools (FI, CFS, EFT, EFS) have a complementary role in the net clinical benefit expected from the procedure. Severe frailty detected through appropriate multidimensional scores (MPI, FI, and CFS) as well as the presence of some specific geriatric conditions such as advanced dementia, severe sarcopenia/cachexia, disability for all/most basic functions of daily living, and poor mobility/bed-bound–should strongly discourage the procedure. 

Assessing frailty indices with the involvement of geriatricians is crucial. Additionally, given the prevalence of CLD, CKD, and other concomitant comorbidities in potential TAVR candidates, the involvement of a pulmonologist, cardiologist, nephrologist, thoracic specialist, HF specialist, nutritionist, physiotherapist, neurologist, as well as other disease specialists, should be considered based on the presence or absence of relevant comorbidities. The help of a MDT may be very beneficial, allowing tailored management of the patients, also taking into account adequate nutrition and proper physical activity [131].

## Figures and Tables

**Table 1:** Median of chronological age in TAVR patients.

Author	Total Patients	Median Age	Male	Female	30-Day Mortality	Stroke	Pacemaker
Carroll, J.D. [29]	276,316	82	149,657 (54.16)	126,627 (45.83)	7980 (3.32)	5009 (1.81)	22,911 (9.98)
Mack, M.J. [6]	1000 496 TAVR/454 SAVR	73.3 ± 5.8	658	292	19/492 * (3.9)	3 (0.6) *	-
Van Mieghem, N.M. [30]	1660 864 TAVR/796 SAVR	79.8 (6.2)		724 (43.6)	-	32.2 *	289 (39.1%) *
Leon, M.B. [4]	2032	81.5 ± 6.7	115/548 * (54.2)		33 (3.3) *	55 (5.5) *	85.5 (8.5) *
Smith, C.R. [7]	699	83.6	281/348 *	-	12 (3.4) *	13 (3.08) *	13 (3.8) *
Kundi, H. et al. [31]	28,531	81.5 (8.1)	15,304 (53.6%)	-	-	99 (0.3)	-
Jørgensen, T.H. [32]	280	79.1 ± 4.8	78 (53.8%)	-	51.8 (8.5) *	8.3 (1.4) *	42.5 (11.0) *

* TAVR group.

**Table 2:** Core components of the CGA.

Domain	Tool	Measure/Score	Time
Comorbidity	Charlson Comorbidity index (CCI) [43]	10-year expected survival	~5 min
	Cumulative Illness Rating Scale (CIRS) [44]	0–56 points (higher score means higher severity)	~5 min
Functional Autonomy	Basic Activities of Daily Living (BADL) [45]	0–6 points (higher score means greater functional dependence)	~3 min
	Instrumental Activities of Daily Living (IADL) [46]	0–8 points (lower score means lower functional autonomy)	~3 min
Cognitive and Mood	Short Portable Mental Status Questionnaire (SPMSQ) [47]	0–10 points (higher score means higher risk of cognitive impairment)	~3 min
	Mini-Mental State Examination (MMSE) [48]	0–30 points (lower score means greater severity of cognitive impairment)	~10 min
	Geriatric Depression Scale (GDS)	0–15 points (higher score means higher severity of depression)	~10 min
Nutritional Status	Mini Nutritional Assessment (MNA) [49]	0–30 points (lower score means higher risk of malnutrition)	~5 min
	Geriatric Nutrition Risk Index (GNRI) [50]	moderate-high risk of hospital complications in patients with score < 92	~5 min
Sarcopenia	EWGSOP2 definition [51]	*probable* with low muscle strength; *confirmed* with low muscle strength + low muscle quantity/quality; *severe* with low muscle strength + low muscle quantity or quality + low physical performance	
Sarcopenia-related	Short Physical Performance Battery [52]	0–12 points (lower score means worse physical performance)	~15 min

Comprehensive Geriatric Assessment (CGA) is a multidimensional and interdisciplinary diagnostic tool used to determine elderlies’ medical, psychosocial, and functional status.

## References

[1] Owens D.S., Bartz T.M., Buzkova P., Massera D., Biggs M.L., Carlson S.D., Psaty B.M., Sotoodehnia N., Gottdiener J.S., Kizer J.R. (2021). Cumulative burden of clinically significant aortic stenosis in community-dwelling older adults. Heart.

[2] Stewart S., Chan Y.K., Playford D., Strange G.A. (2022). Incident aortic stenosis in 49,449 men and 42,229 women investigated with routine echocardiography. Heart.

[3] Kolkailah A.A., Doukky R., Pelletier M.P., Volgman A.S., Kaneko T., Nabhan A.F. (2020). Cochrane corner: Transcatheter aortic valve implantation versus surgical aortic valve replacement for severe aortic stenosis in people with low surgical risk. Heart.

[4] Leon M.B., Smith C.R., Mack M.J., Makkar R.R., Svensson L.G., Kodali S.K., Thourani V.H., Tuzcu E.M., Miller D.C., Herrmann H.C. (2016). Transcatheter or Surgical Aortic-Valve Replacement in Intermediate-Risk Patients. N. Engl. J. Med..

[5] Leon M.B., Smith C.R., Mack M., Miller D.C., Moses J.W., Svensson L.G., Tuzcu E.M., Webb J.G., Fontana G.P., Makkar R.R. (2010). Transcatheter aortic-valve implantation for aortic stenosis in patients who cannot undergo surgery. N. Engl. J. Med..

[6] Mack M.J., Leon M.B., Thourani V.H., Makkar R., Kodali S.K., Russo M., Kapadia S.R., Malaisrie S.C., Cohen D.J., Pibarot P. (2019). Transcatheter Aortic-Valve Replacement with a Balloon-Expandable Valve in Low-Risk Patients. N. Engl. J. Med..

[7] Smith C.R., Leon M.B., Mack M.J., Miller D.C., Moses J.W., Svensson L.G., Tuzcu E.M., Webb J.G., Fontana G.P., Makkar R.R. (2011). Transcatheter versus surgical aortic-valve replacement in high-risk patients. N. Engl. J. Med..

[8] Bobet A.S., Brouessard C., Le Tourneau T., Manigold T., de Decker L., Boureau A.S. (2022). Length of Stay in Older Patients Undergoing Transcatheter Aortic Valve Replacement: Value of a Geriatric Approach. Gerontology.

[9] Kim D.H., Afilalo J., Shi S.M., Popma J.J., Khabbaz K.R., Laham R.J., Grodstein F., Guibone K., Lux E., Lipsitz L.A. (2019). Evaluation of Changes in Functional Status in the Year after Aortic Valve Replacement. JAMA Intern. Med..

[10] Lindman B.R., Alexander K.P., O’Gara P.T., Afilalo J. (2014). Futility, benefit, and transcatheter aortic valve replacement. JACC Cardiovasc. Interv..

[11] Damluji A.A., Bernacki G., Afilalo J., Lyubarova R., Orkaby A.R., Kwak M.J., Hummel S., Kirkpatrick J.N., Maurer M.S., Wenger N. (2024). TAVR in Older Adults: Moving toward a Comprehensive Geriatric Assessment and Away from Chronological Age: JACC Family Series. JACC Adv..

[12] Biancari F., Juvonen T., Onorati F., Faggian G., Heikkinen J., Airaksinen J., Mariscalco G. (2014). Meta-analysis on the performance of the EuroSCORE II and the Society of Thoracic Surgeons Scores in patients undergoing aortic valve replacement. J. Cardiothorac. Vasc. Anesth..

[13] Martin G.P., Sperrin M., Ludman P.F., de Belder M.A., Gale C.P., Toff W.D., Moat N.E., Trivedi U., Buchan I., Mamas M.A. (2017). Inadequacy of existing clinical prediction models for predicting mortality after transcatheter aortic valve implantation. Am. Heart J..

[14] Coylewright M., Palmer R., O’Neill E.S., Robb J.F., Fried T.R. (2016). Patient-defined goals for the treatment of severe aortic stenosis: A qualitative analysis. Health Expect..

[15] Delijani D., Li L., Rutkin B., Wilson S., Kennedy K.F., Hartman A.R., Yu P.J. (2023). Impact of age on outcomes after transcatheter aortic valve implantation. Eur. Heart J. Qual. Care Clin. Outcomes.

[16] Stehli J., Koh J.Q.S., Duffy S.J., Zamani J., Yeong C.C., Paratz E., Martin C., Htun N.M., Stub D., Dick R. (2019). Comparison of Outcomes of Transcatheter Aortic Valve Implantation in Patients Aged >90 Years versus <90 Years. Am. J. Cardiol..

[17] Fragasso G. (2016). ‘Heart Team’ decision-making for cardiac interventional procedures should take into account patients’ cognitive function and frailty. J. Cardiovasc. Med..

[18] Mazza A., Iafrancesco M., Bruno P., Chiariello G.A., Trani C., Burzotta F., Cammertoni F., Pasquini A., Diana G., Rosenhek R. (2023). The multidisciplinary Heart Team approach for patients with cardiovascular disease: A step towards personalized medicine. J. Cardiovasc. Med..

[19] Pagnesi M., Adamo M., Sama I.E., Anker S.D., Cleland J.G., Dickstein K., Filippatos G.S., Lang C.C., Ng L.L., Ponikowski P. (2021). Impact of mitral regurgitation in patients with worsening heart failure: Insights from BIOSTAT-CHF. Eur. J. Heart Fail..

[20] Riccardi M., Cikes M., Adamo M., Pagnesi M., Lombardi C.M., Solomon S.D., Metra M., Inciardi R.M. (2024). Functional Mitral Regurgitation and Heart Failure with Preserved Ejection Fraction: Clinical Implications and Management. J. Card. Fail..

[21] Lucà F., Oliva F., Abrignani M.G., Di Fusco S.A., Gori M., Giubilato S., Ceravolo R., Temporelli P.L., Cornara S., Rao C.M. (2024). Heart Failure with Preserved Ejection Fraction: How to Deal with This Chameleon. J. Clin. Med..

[22] Jabbour A., Ismail T.F., Moat N., Gulati A., Roussin I., Alpendurada F., Park B., Okoroafor F., Asgar A., Barker S. (2011). Multimodality imaging in transcatheter aortic valve implantation and post-procedural aortic regurgitation: Comparison among cardiovascular magnetic resonance, cardiac computed tomography, and echocardiography. J. Am. Coll. Cardiol..

[23] Lucà F., Abrignani M.G., Parrini I., Di Fusco S.A., Giubilato S., Rao C.M., Piccioni L., Cipolletta L., Passaretti B., Giallauria F. (2022). Update on Management of Cardiovascular Diseases in Women. J. Clin. Med..

[24] Lucà F., Pavan D., Gulizia M.M., Manes M.T., Abrignani M.G., Benedetto F.A., Bisceglia I., Brigido S., Caldarola P., Calvanese R. (2024). Italian Association of Hospital Cardiologists Position Paper ‘Gender discrepancy: Time to implement gender-based clinical management’. Eur. Heart J. Suppl..

[25] Lucà F., Pavan D., Gulizia M.M., Manes M.T., Abrignani M.G., Benedetto F.A., Bisceglia I., Brigido S., Caldarola P., Calvanese R. (2024). Position paper ANMCO: Differenze di genere nell’approccio farmacologico cardiovascolare. G. Ital. Di Cardiol..

[26] Hariri E., Yang E., Whelton S.P. (2023). Personalizing Cardiovascular Disease Risk Assessment. JACC Asia.

[27] Hamczyk M.R., Nevado R.M., Barettino A., Fuster V., Andrés V. (2020). Biological versus Chronological Aging: JACC Focus Seminar. J. Am. Coll. Cardiol..

[28] Diebel L.W.M., Rockwood K. (2021). Determination of Biological Age: Geriatric Assessment vs Biological Biomarkers. Curr. Oncol. Rep..

[29] Carroll J.D., Mack M.J., Vemulapalli S., Herrmann H.C., Gleason T.G., Hanzel G., Deeb G.M., Thourani V.H., Cohen D.J., Desai N. (2020). STS-ACC TVT Registry of Transcatheter Aortic Valve Replacement. J. Am. Coll. Cardiol..

[30] Van Mieghem N.M., Deeb G.M., Søndergaard L., Grube E., Windecker S., Gada H., Mumtaz M., Olsen P.S., Heiser J.C., Merhi W. (2022). Self-expanding Transcatheter vs Surgical Aortic Valve Replacement in Intermediate-Risk Patients: 5-Year Outcomes of the SURTAVI Randomized Clinical Trial. JAMA Cardiol..

[31] Kundi H., Popma J.J., Reynolds M.R., Strom J.B., Pinto D.S., Valsdottir L.R., Shen C., Choi E., Yeh R.W. (2019). Frailty and related outcomes in patients undergoing transcatheter valve therapies in a nationwide cohort. Eur. Heart J..

[32] Jørgensen T.H., Thyregod H.G.H., Ihlemann N., Nissen H., Petursson P., Kjeldsen B.J., Steinbrüchel D.A., Olsen P.S., Søndergaard L. (2021). Eight-year outcomes for patients with aortic valve stenosis at low surgical risk randomized to transcatheter vs. surgical aortic valve replacement. Eur. Heart J..

[33] Gelsomino S., Lucà F., Parise O., Lorusso R., Rao C.M., Vizzardi E., Gensini G.F., Maessen J.G. (2013). Longitudinal strain predicts left ventricular mass regression after aortic valve replacement for severe aortic stenosis and preserved left ventricular function. Heart Vessel..

[34] Sundt T.M., Jneid H. (2021). Guideline Update on Indications for Transcatheter Aortic Valve Implantation Based on the 2020 American College of Cardiology/American Heart Association Guidelines for Management of Valvular Heart Disease. JAMA Cardiol..

[35] Vahanian A., Beyersdorf F., Praz F., Milojevic M., Baldus S., Bauersachs J., Capodanno D., Conradi L., De Bonis M., De Paulis R. (2021). 2021 ESC/EACTS Guidelines for the management of valvular heart disease: Developed by the Task Force for the management of valvular heart disease of the European Society of Cardiology (ESC) and the European Association for Cardio-Thoracic Surgery (EACTS). Eur. Heart J..

[36] Vendrik J., van Mourik M.S., van Kesteren F., Henstra M.J., Piek J.J., Henriques J.P.S., Wykrzykowska J.J., de Winter R.J., Vis M.M., Koch K.T. (2018). Comparison of Outcomes of Transfemoral Aortic Valve Implantation in Patients <90 with Those >90 Years of Age. Am. J. Cardiol..

[37] Vlastra W., Chandrasekhar J., Vendrik J., Gutierrez-Ibanes E., Tchétché D., de Brito F.S., Barbanti M., Kornowski R., Latib A., D’Onofrio A. (2019). Transfemoral TAVR in Nonagenarians: From the CENTER Collaboration. JACC Cardiovasc. Interv..

[38] Murthi M., Velagapudi S., Sharma B., Ezegwu O., Akuna E., Park D.Y., Atluri R., Vardar U. (2022). Comparison of In-Hospital Mortality and Clinical Outcomes Between Patients Aged More Than and Less Than 80 Years Undergoing Transcatheter Aortic Valve Replacement. Cureus.

[39] Arsalan M., Szerlip M., Vemulapalli S., Holper E.M., Arnold S.V., Li Z., DiMaio M.J., Rumsfeld J.S., Brown D.L., Mack M.J. (2016). Should Transcatheter Aortic Valve Replacement Be Performed in Nonagenarians?: Insights from the STS/ACC TVT Registry. J. Am. Coll. Cardiol..

[40] Chiarito M., Spirito A., Nicolas J., Selberg A., Stefanini G., Colombo A., Reimers B., Kini A., Sharma S.K., Dangas G.D. (2022). Evolving Devices and Material in Transcatheter Aortic Valve Replacement: What to Use and for Whom. J. Clin. Med..

[41] Cruz-Jentoft A.J., Daragjati J., Fratiglioni L., Maggi S., Mangoni A.A., Mattace-Raso F., Paccalin M., Polidori M.C., Topinkova E., Ferrucci L. (2020). Using the Multidimensional Prognostic Index (MPI) to improve cost-effectiveness of interventions in multimorbid frail older persons: Results and final recommendations from the MPI_AGE European Project. Aging Clin. Exp. Res..

[42] Stuck A.E., Siu A.L., Wieland G.D., Adams J., Rubenstein L.Z. (1993). Comprehensive geriatric assessment: A meta-analysis of controlled trials. Lancet.

[43] Charlson M.E., Pompei P., Ales K.L., MacKenzie C.R. (1987). A new method of classifying prognostic comorbidity in longitudinal studies: Development and validation. J. Chronic Dis..

[44] Parmelee P.A., Thuras P.D., Katz I.R., Lawton M.P. (1995). Validation of the Cumulative Illness Rating Scale in a geriatric residential population. J. Am. Geriatr. Soc..

[45] Katz S., Downs T.D., Cash H.R., Grotz R.C. (1970). Progress in development of the index of ADL. Gerontologist.

[46] Lawton M.P., Brody E.M. (1969). Assessment of older people: Self-maintaining and instrumental activities of daily living. Gerontologist.

[47] Pfeiffer E. (1975). A short portable mental status questionnaire for the assessment of organic brain deficit in elderly patients. J. Am. Geriatr. Soc..

[48] Folstein M.F., Folstein S.E., McHugh P.R. (1975). “Mini-mental state”. A practical method for grading the cognitive state of patients for the clinician. J. Psychiatr. Res..

[49] Guigoz Y. (2006). The Mini Nutritional Assessment (MNA) review of the literature—What does it tell us?. J. Nutr. Health Aging.

[50] Bouillanne O., Morineau G., Dupont C., Coulombel I., Vincent J.P., Nicolis I., Benazeth S., Cynober L., Aussel C. (2005). Geriatric Nutritional Risk Index: A new index for evaluating at-risk elderly medical patients. Am. J. Clin. Nutr..

[51] Cruz-Jentoft A.J., Bahat G., Bauer J., Boirie Y., Bruyère O., Cederholm T., Cooper C., Landi F., Rolland Y., Sayer A.A. (2019). Sarcopenia: Revised European consensus on definition and diagnosis. Age Ageing.

[52] Guralnik J.M., Simonsick E.M., Ferrucci L., Glynn R.J., Berkman L.F., Blazer D.G., Scherr P.A., Wallace R.B. (1994). A short physical performance battery assessing lower extremity function: Association with self-reported disability and prediction of mortality and nursing home admission. J. Gerontol..

[53] Reuben D.B., Borok G.M., Wolde-Tsadik G., Ershoff D.H., Fishman L.K., Ambrosini V.L., Liu Y., Rubenstein L.Z., Beck J.C. (1995). A randomized trial of comprehensive geriatric assessment in the care of hospitalized patients. N. Engl. J. Med..

[54] Welsh T.J., Gordon A.L., Gladman J.R. (2014). Comprehensive geriatric assessment—A guide for the non-specialist. Int. J. Clin. Pract..

[55] Beard J.R., Officer A., de Carvalho I.A., Sadana R., Pot A.M., Michel J.P., Lloyd-Sherlock P., Epping-Jordan J.E., Peeters G., Mahanani W.R. (2016). The World report on ageing and health: A policy framework for healthy ageing. Lancet.

[56] Rozzini R., Bianchetti A., Alboni P., Baldasseroni S., Bo M., Boccanelli A., Desideri G., Marchionni N., Palazzo G., Terrosu P. (2022). The older patient with cardiovascular disease: Background and clinical implications of the comprehensive geriatric assessment. Minerva Med..

[57] Sündermann S.H., Bäck C., Bischoff-Ferrari H.A., Dehbi H.M., Szekely A., Völler H., Niebauer J. (2023). Preinterventional frailty assessment in patients scheduled for cardiac surgery or transcatheter aortic valve implantation: A consensus statement of the European Association for Cardio-Thoracic Surgery (EACTS) and the European Association of Preventive Cardiology (EAPC) of the European Society of Cardiology (ESC). Eur. J. Cardiothorac. Surg..

[58] Andò G., Basile G. (2020). Sarcopenia: Only one of the domains of frailty in patients undergoing transcatheter aortic valve implantation. J. Cardiovasc. Med..

[59] Stortecky S., Schoenenberger A.W., Moser A., Kalesan B., Jüni P., Carrel T., Bischoff S., Schoenenberger C.M., Stuck A.E., Windecker S. (2012). Evaluation of multidimensional geriatric assessment as a predictor of mortality and cardiovascular events after transcatheter aortic valve implantation. JACC Cardiovasc. Interv..

[60] Ungar A., Mannarino G., van der Velde N., Baan J., Thibodeau M.P., Masson J.B., Santoro G., van Mourik M., Jansen S., Deutsch C. (2018). Comprehensive geriatric assessment in patients undergoing transcatheter aortic valve implantation—Results from the CGA-TAVI multicentre registry. BMC Cardiovasc. Disord..

[61] Bo M., Bergamo D., Calvi E., Iacovino M., Falcone Y., Grisoglio E., Salizzoni S. (2020). Role of comprehensive geriatric assessment in low surgical risk older patients with aortic stenosis. Aging Clin. Exp. Res..

[62] Boureau A.S., Trochu J.N., Colliard C., Volteau C., Jaafar P., Manigold T., Le Tourneau T., Berrut G., de Decker L. (2015). Determinants in treatment decision-making in older patients with symptomatic severe aortic stenosis. Maturitas.

[63] Schäfer M., Körber M.I., Vimalathasan R., Mauri V., Iliadis C., Metze C., Ten Freyhaus H., Baldus S., Polidori M.C., Pfister R. (2021). Risk Stratification of Patients Undergoing Percutaneous Repair of Mitral and Tricuspid Valves Using a Multidimensional Geriatric Assessment. Circ. Cardiovasc. Qual. Outcomes.

[64] Sá M.P., Erten O., Ramlawi B. (2022). Transcatheter Aortic Valve Implantation in Elderly Patients with Aortic Valve Stenosis: The Role of Frailty, Malnutrition, and Sarcopenia. J. Am. Heart Assoc..

[65] Yu Z., Zhao Q., Ye Y., Wang M., Zhou Z., Zhang H., Zhao Z., Liu Q., Zhang Z., Wu Y. (2021). Comprehensive Geriatric Assessment and Exercise Capacity in Cardiac Rehabilitation for Patients Referred to Transcatheter Aortic Valve Implantation. Am. J. Cardiol..

[66] Goudzwaard J.A., Chotkan S., De Ronde-Tillmans M., Lenzen M.J., van Wiechen M.P.H., Ooms J.F.W., Polinder-Bos H.A., de Beer-Leentfaar M., Van Mieghem N.M., Daemen J. (2021). Multidimensional Prognostic Index and Outcomes in Older Patients Undergoing Transcatheter Aortic Valve Implantation: Survival of the Fittest. J. Clin. Med..

[67] Asgar A.W., Ouzounian M., Adams C., Afilalo J., Fremes S., Lauck S., Leipsic J., Piazza N., Rodes-Cabau J., Welsh R. (2019). 2019 Canadian Cardiovascular Society Position Statement for Transcatheter Aortic Valve Implantation. Can. J. Cardiol..

[68] Goudzwaard J.A., de Ronde-Tillmans M., van Hoorn F.E.D., Kwekkeboom E.H.C., Lenzen M.J., van Wiechen M.P.H., Ooms J.F.W., Nuis R.J., Van Mieghem N.M., Daemen J. (2020). Impact of frailty on health-related quality of life 1 year after transcatheter aortic valve implantation. Age Ageing.

[69] Zucchelli A., Manzoni F., Morandi A., Di Santo S., Rossi E., Valsecchi M.G., Inzitari M., Cherubini A., Bo M., Mossello E. (2022). The association between low skeletal muscle mass and delirium: Results from the nationwide multi-centre Italian Delirium Day 2017. Aging Clin. Exp. Res..

[70] Bellelli G., Zambon A., Volpato S., Abete P., Bianchi L., Bo M., Cherubini A., Corica F., Di Bari M., Maggio M. (2018). The association between delirium and sarcopenia in older adult patients admitted to acute geriatrics units: Results from the GLISTEN multicenter observational study. Clin. Nutr..

[71] Hosler Q.P., Maltagliati A.J., Shi S.M., Afilalo J., Popma J.J., Khabbaz K.R., Laham R.J., Guibone K., Kim D.H. (2019). A Practical Two-Stage Frailty Assessment for Older Adults Undergoing Aortic Valve Replacement. J. Am. Geriatr. Soc..

[72] Dent E., Martin F.C., Bergman H., Woo J., Romero-Ortuno R., Walston J.D. (2019). Management of frailty: Opportunities, challenges, and future directions. Lancet.

[73] Presta R., Brunetti E., Polidori M.C., Bo M. (2022). Impact of frailty models on the prescription of oral anticoagulants and on the incidence of stroke, bleeding, and mortality in older patients with atrial fibrillation: A systematic review. Ageing Res. Rev..

[74] Nishimura R.A., Otto C.M., Bonow R.O., Carabello B.A., Erwin J.P., Fleisher L.A., Jneid H., Mack M.J., McLeod C.J., O’Gara P.T. (2017). 2017 AHA/ACC Focused Update of the 2014 AHA/ACC Guideline for the Management of Patients with Valvular Heart Disease: A Report of the American College of Cardiology/American Heart Association Task Force on Clinical Practice Guidelines. Circulation.

[75] Baumgartner H., Falk V., Bax J.J., De Bonis M., Hamm C., Holm P.J., Iung B., Lancellotti P., Lansac E., Muñoz D.R. (2018). 2017 ESC/EACTS Guidelines for the Management of Valvular Heart Disease. Rev. Esp. Cardiol..

[76] Fried L.P., Tangen C.M., Walston J., Newman A.B., Hirsch C., Gottdiener J., Seeman T., Tracy R., Kop W.J., Burke G. (2001). Frailty in older adults: Evidence for a phenotype. J. Gerontol. A Biol. Sci. Med. Sci..

[77] Mitnitski A.B., Mogilner A.J., Rockwood K. (2001). Accumulation of deficits as a proxy measure of aging. Sci. World J..

[78] Jones D.M., Song X., Rockwood K. (2004). Operationalizing a frailty index from a standardized comprehensive geriatric assessment. J. Am. Geriatr. Soc..

[79] Rockwood K., Song X., MacKnight C., Bergman H., Hogan D.B., McDowell I., Mitnitski A. (2005). A global clinical measure of fitness and frailty in elderly people. Can. Med. Assoc. J..

[80] Rockwood K., Theou O. (2020). Using the Clinical Frailty Scale in Allocating Scarce Health Care Resources. Can. Geriatr. J..

[81] Shrier W., Dewar C., Parrella P., Hunt D., Hodgson L.E. (2021). Agreement and predictive value of the Rockwood Clinical Frailty Scale at emergency department triage. Emerg. Med. J..

[82] Afilalo J., Alexander K.P., Mack M.J., Maurer M.S., Green P., Allen L.A., Popma J.J., Ferrucci L., Forman D.E. (2014). Frailty assessment in the cardiovascular care of older adults. J. Am. Coll. Cardiol..

[83] Strom J.B., Xu J., Orkaby A.R., Shen C., Song Y., Charest B.R., Kim D.H., Cohen D.J., Kramer D.B., Spertus J.A. (2021). Role of Frailty in Identifying Benefit From Transcatheter versus Surgical Aortic Valve Replacement. Circ. Cardiovasc. Qual. Outcomes.

[84] Rodríguez-Pascual C., Paredes-Galán E., Ferrero-Martínez A.I., Baz-Alonso J.A., Durán-Muñoz D., González-Babarro E., Sanmartín M., Parajes T., Torres-Torres I., Piñón-Esteban M. (2016). The frailty syndrome and mortality among very old patients with symptomatic severe aortic stenosis under different treatments. Int. J. Cardiol..

[85] Huded C.P., Huded J.M., Friedman J.L., Benck L.R., Lindquist L.A., Holly T.A., Sweis R.N., Ricciardi M.J., Malaisrie S.C., Davidson C.J. (2016). Frailty Status and Outcomes after Transcatheter Aortic Valve Implantation. Am. J. Cardiol..

[86] Shimura T., Yamamoto M., Kano S., Kagase A., Kodama A., Koyama Y., Tsuchikane E., Suzuki T., Otsuka T., Kohsaka S. (2017). Impact of the Clinical Frailty Scale on Outcomes after Transcatheter Aortic Valve Replacement. Circulation.

[87] Afilalo J., Lauck S., Kim D.H., Lefèvre T., Piazza N., Lachapelle K., Martucci G., Lamy A., Labinaz M., Peterson M.D. (2017). Frailty in Older Adults Undergoing Aortic Valve Replacement: The FRAILTY-AVR Study. J. Am. Coll. Cardiol..

[88] Mazzola P., Tassistro E., Di Santo S., Rossi E., Andreano A., Valsecchi M.G., Cherubini A., Marengoni A., Mossello E., Bo M. (2021). The relationship between frailty and delirium: Insights from the 2017 Delirium Day study. Age Ageing.

[89] Shi S., Afilalo J., Lipsitz L.A., Popma J.J., Khabbaz K.R., Laham R.J., Guibone K., Grodstein F., Lux E., Kim D.H. (2019). Frailty Phenotype and Deficit Accumulation Frailty Index in Predicting Recovery after Transcatheter and Surgical Aortic Valve Replacement. J. Gerontol. A Biol. Sci. Med. Sci..

[90] Goldfarb M., Bendayan M., Rudski L.G., Morin J.F., Langlois Y., Ma F., Lachapelle K., Cecere R., DeVarennes B., Tchervenkov C.I. (2017). Cost of Cardiac Surgery in Frail Compared with Nonfrail Older Adults. Can. J. Cardiol..

[91] Kiani S., Stebbins A., Thourani V.H., Forcillo J., Vemulapalli S., Kosinski A.S., Babaliaros V., Cohen D., Kodali S.K., Kirtane A.J. (2020). The Effect and Relationship of Frailty Indices on Survival after Transcatheter Aortic Valve Replacement. JACC Cardiovasc. Interv..

[92] Zusman O., Kornowski R., Witberg G., Lador A., Orvin K., Levi A., Assali A., Vaknin-Assa H., Sharony R., Shapira Y. (2017). Transcatheter Aortic Valve Implantation Futility Risk Model Development and Validation among Treated Patients with Aortic Stenosis. Am. J. Cardiol..

[93] Arnold S.V., Cohen D.J., Dai D., Jones P.G., Li F., Thomas L., Baron S.J., Frankel N.Z., Strong S., Matsouaka R.A. (2018). Predicting Quality of Life at 1 Year after Transcatheter Aortic Valve Replacement in a Real-World Population. Circ. Cardiovasc. Qual. Outcomes.

[94] Arnold S.V., Spertus J.A., Lei Y., Green P., Kirtane A.J., Kapadia S., Thourani V.H., Herrmann H.C., Beohar N., Zajarias A. (2013). How to define a poor outcome after transcatheter aortic valve replacement: Conceptual framework and empirical observations from the placement of aortic transcatheter valve (PARTNER) trial. Circ. Cardiovasc. Qual. Outcomes.

[95] Puri R., Iung B., Cohen D.J., Rodés-Cabau J. (2016). TAVI or No TAVI: Identifying patients unlikely to benefit from transcatheter aortic valve implantation. Eur. Heart J..

[96] Chopard R., Meneveau N., Chocron S., Gilard M., Laskar M., Eltchaninoff H., Iung B., Leprince P., Teiger E., Chevreul K. (2014). Impact of chronic obstructive pulmonary disease on Valve Academic Research Consortium-defined outcomes after transcatheter aortic valve implantation (from the FRANCE 2 Registry). Am. J. Cardiol..

[97] Ludman P.F., Moat N., de Belder M.A., Blackman D.J., Duncan A., Banya W., MacCarthy P.A., Cunningham D., Wendler O., Marlee D. (2015). Transcatheter aortic valve implantation in the United Kingdom: Temporal trends, predictors of outcome, and 6-year follow-up: A report from the UK Transcatheter Aortic Valve Implantation (TAVI) Registry, 2007 to 2012. Circulation.

[98] Holmes D.R., Brennan J.M., Rumsfeld J.S., Dai D., O’Brien S.M., Vemulapalli S., Edwards F.H., Carroll J., Shahian D., Grover F. (2015). Clinical outcomes at 1 year following transcatheter aortic valve replacement. JAMA.

[99] Yamamoto M., Hayashida K., Mouillet G., Hovasse T., Chevalier B., Oguri A., Watanabe Y., Dubois-Randé J.L., Morice M.C., Lefèvre T. (2013). Prognostic value of chronic kidney disease after transcatheter aortic valve implantation. J. Am. Coll. Cardiol..

[100] Dumonteil N., van der Boon R.M., Tchetche D., Chieffo A., Van Mieghem N.M., Marcheix B., Buchanan G.L., Vahdat O., Serruys P.W., Fajadet J. (2013). Impact of preoperative chronic kidney disease on short-and long-term outcomes after transcatheter aortic valve implantation: A Pooled-RotterdAm-Milano-Toulouse In Collaboration Plus (PRAGMATIC-Plus) initiative substudy. Am. Heart J..

[101] Lee Z.X., Elangovan S., Anderson R., Groves P. (2020). Short- and medium-term survival after TAVI: Clinical predictors and the role of the FRANCE-2 score. Int. J. Cardiol. Heart Vasc..

[102] Allende R., Webb J.G., Munoz-Garcia A.J., de Jaegere P., Tamburino C., Dager A.E., Cheema A., Serra V., Amat-Santos I., Velianou J.L. (2014). Advanced chronic kidney disease in patients undergoing transcatheter aortic valve implantation: Insights on clinical outcomes and prognostic markers from a large cohort of patients. Eur. Heart J..

[103] Barbash I.M., Escarcega R.O., Minha S., Ben-Dor I., Torguson R., Goldstein S.A., Wang Z., Okubagzi P., Satler L.F., Pichard A.D. (2015). Prevalence and impact of pulmonary hypertension on patients with aortic stenosis who underwent transcatheter aortic valve replacement. Am. J. Cardiol..

[104] Maeder M.T., Weber L., Weilenmann D., Haager P.K., Joerg L., Rohner F., Ammann P., Chronis J., Rigger J., Rickli H. (2021). Impact of a volume challenge on haemodynamics and prognosis in patients with severe aortic stenosis. ESC Heart Fail..

[105] Himbert D., Vahanian A. (2015). Transcatheter aortic valve replacement for patients with heart failure. Heart Fail. Clin..

[106] Tamburino C., Capodanno D., Ramondo A., Petronio A.S., Ettori F., Santoro G., Klugmann S., Bedogni F., Maisano F., Marzocchi A. (2011). Incidence and predictors of early and late mortality after transcatheter aortic valve implantation in 663 patients with severe aortic stenosis. Circulation.

[107] Urena M., Webb J.G., Eltchaninoff H., Muñoz-García A.J., Bouleti C., Tamburino C., Nombela-Franco L., Nietlispach F., Moris C., Ruel M. (2015). Late cardiac death in patients undergoing transcatheter aortic valve replacement: Incidence and predictors of advanced heart failure and sudden cardiac death. J. Am. Coll. Cardiol..

[108] Elmariah S., Palacios I.F., McAndrew T., Hueter I., Inglessis I., Baker J.N., Kodali S., Leon M.B., Svensson L., Pibarot P. (2013). Outcomes of transcatheter and surgical aortic valve replacement in high-risk patients with aortic stenosis and left ventricular dysfunction: Results from the Placement of Aortic Transcatheter Valves (PARTNER) trial (cohort A). Circ. Cardiovasc. Interv..

[109] Herrmann H.C., Pibarot P., Hueter I., Gertz Z.M., Stewart W.J., Kapadia S., Tuzcu E.M., Babaliaros V., Thourani V., Szeto W.Y. (2013). Predictors of mortality and outcomes of therapy in low-flow severe aortic stenosis: A Placement of Aortic Transcatheter Valves (PARTNER) trial analysis. Circulation.

[110] Nombela-Franco L., Ribeiro H.B., Urena M., Allende R., Amat-Santos I., DeLarochellière R., Dumont E., Doyle D., DeLarochellière H., Laflamme J. (2014). Significant mitral regurgitation left untreated at the time of aortic valve replacement: A comprehensive review of a frequent entity in the transcatheter aortic valve replacement era. J. Am. Coll. Cardiol..

[111] Toggweiler S., Boone R.H., Rodés-Cabau J., Humphries K.H., Lee M., Nombela-Franco L., Bagur R., Willson A.B., Binder R.K., Gurvitch R. (2012). Transcatheter aortic valve replacement: Outcomes of patients with moderate or severe mitral regurgitation. J. Am. Coll. Cardiol..

[112] Barbanti M., Webb J.G., Hahn R.T., Feldman T., Boone R.H., Smith C.R., Kodali S., Zajarias A., Thompson C.R., Green P. (2013). Impact of preoperative moderate/severe mitral regurgitation on 2-year outcome after transcatheter and surgical aortic valve replacement: Insight from the Placement of Aortic Transcatheter Valve (PARTNER) Trial Cohort A. Circulation.

[113] Nombela-Franco L., Eltchaninoff H., Zahn R., Testa L., Leon M.B., Trillo-Nouche R., D’Onofrio A., Smith C.R., Webb J., Bleiziffer S. (2015). Clinical impact and evolution of mitral regurgitation following transcatheter aortic valve replacement: A meta-analysis. Heart.

[114] Otto C.M., Kumbhani D.J., Alexander K.P., Calhoon J.H., Desai M.Y., Kaul S., Lee J.C., Ruiz C.E., Vassileva C.M. (2017). 2017 ACC Expert Consensus Decision Pathway for Transcatheter Aortic Valve Replacement in the Management of Adults with Aortic Stenosis: A Report of the American College of Cardiology Task Force on Clinical Expert Consensus Documents. J. Am. Coll. Cardiol..

[115] Afilalo J. (2017). The Clinical Frailty Scale: Upgrade Your Eyeball Test. Circulation.

[116] Paolisso P., Beles M., Belmonte M., Gallinoro E., De Colle C., Mileva N., Bertolone D.T., Deschepper C., Spapen J., Brouwers S. (2023). Outcomes in patients with moderate and asymptomatic severe aortic stenosis followed up in heart valve clinics. Heart.

[117] Lancellotti P., Magne J., Dulgheru R., Clavel M.A., Donal E., Vannan M.A., Chambers J., Rosenhek R., Habib G., Lloyd G. (2018). Outcomes of Patients with Asymptomatic Aortic Stenosis Followed Up in Heart Valve Clinics. JAMA Cardiol..

[118] Lim W.Y., Ramasamy A., Lloyd G., Bhattacharyya S. (2017). Meta-analysis of the impact of intervention versus symptom-driven management in asymptomatic severe aortic stenosis. Heart.

[119] Zilberszac R., Lancellotti P., Gilon D., Gabriel H., Schemper M., Maurer G., Massetti M., Rosenhek R. (2017). Role of a heart valve clinic programme in the management of patients with aortic stenosis. Eur. Heart J. Cardiovasc. Imaging.

[120] Kvidal P., Bergström R., Hörte L.G., Ståhle E. (2000). Observed and relative survival after aortic valve replacement. J. Am. Coll. Cardiol..

[121] Chioncel O., Adamo M., Nikolaou M., Parissis J., Mebazaa A., Yilmaz M.B., Hassager C., Moura B., Bauersachs J., Harjola V.P. (2023). Acute heart failure and valvular heart disease: A scientific statement of the Heart Failure Association, the Association for Acute CardioVascular Care and the European Association of Percutaneous Cardiovascular Interventions of the European Society of Cardiology. Eur. J. Heart Fail..

[122] Chambers J.B., Lancellotti P. (2020). Heart Valve Clinics, Centers, and Networks. Cardiol. Clin..

[123] Durand E., Borz B., Godin M., Tron C., Litzler P.Y., Bessou J.P., Dacher J.N., Bauer F., Cribier A., Eltchaninoff H. (2013). Performance analysis of EuroSCORE II compared to the original logistic EuroSCORE and STS scores for predicting 30-day mortality after transcatheter aortic valve replacement. Am. J. Cardiol..

[124] van Mieghem N.M., Head S.J., van der Boon R.M., Piazza N., de Jaegere P.P., Carrel T., Kappetein A.P., Lange R., Walther T., Windecker S. (2012). The SURTAVI model: Proposal for a pragmatic risk stratification for patients with severe aortic stenosis. EuroIntervention.

[125] Otto C.M., Nishimura R.A., Bonow R.O., Carabello B.A., Erwin J.P., Gentile F., Jneid H., Krieger E.V., Mack M., McLeod C. (2021). 2020 ACC/AHA Guideline for the Management of Patients with Valvular Heart Disease: Executive Summary: A Report of the American College of Cardiology/American Heart Association Joint Committee on Clinical Practice Guidelines. Circulation.

[126] Postolache A., Sperlongano S., Lancellotti P. (2023). TAVI after More Than 20 Years. J. Clin. Med..

[127] Lucà F., Oliva F., Abrignani M.G., Di Fusco S.A., Parrini I., Canale M.L., Giubilato S., Cornara S., Nesti M., Rao C.M. (2023). Management of Patients Treated with Direct Oral Anticoagulants in Clinical Practice and Challenging Scenarios. J. Clin. Med..

[128] Strange J.E., Christensen D.M., Sindet-Pedersen C., Schou M., Falkentoft A.C., Østergaard L., Butt J.H., Graversen P.L., Køber L., Gislason G. (2023). Frailty and Recurrent Hospitalization after Transcatheter Aortic Valve Replacement. J. Am. Heart Assoc..

[129] O’Neill D., Forman D.E. (2019). Never Too Old for Cardiac Rehabilitation. Clin. Geriatr. Med..

[130] Parrini I., Lucà F., Rao C.M., Cacciatore S., Riccio C., Grimaldi M., Gulizia M.M., Oliva F., Andreotti F. (2024). How to Manage Beta-Blockade in Older Heart Failure Patients: A Scoping Review. J. Clin. Med..

[131] Massussi M., Adamo M., Rosato S., Seccareccia F., Barbanti M., Biancari F., Tarantini G., Immè S., Vignali L., Badoni G. (2022). Functional and metabolic frailty predicts mortality in patients undergoing TAVI: Insights from the OBSERVANT II study. Eur. J. Intern. Med..

